# Clinical Utility of a Structured Program to Reduce the Risk of Health-Related Quality of Life Impairment after Discharge from Intensive Care Unit: A Real-World Experience

**DOI:** 10.1155/2018/3838962

**Published:** 2018-05-08

**Authors:** Angelica Venni, Francesca Ioia, Silvia Laviola, Francesca Frigieri, Alessandra Pieri, Simona Marilli, Daniela Balzi, Piercarlo Ballo, Stefano Gori, Diletta Guarducci

**Affiliations:** ^1^Department of Intensive Care, S. Maria Annunziata Hospital, Florence, Italy; ^2^Department of Epidemiology, Local Health Authority, Florence, Italy; ^3^Department of Cardiology, S. Maria Annunziata Hospital, Florence, Italy

## Abstract

**Background:**

Postdischarge deterioration in health-related quality of life (HRQoL) is a major clinical issue for patients after an intensive care unit (ICU) hospitalization. A significant proportion of these patients is known to develop a progressive worsening of mental and physical performance—the so-called post-intensive care syndrome (PICS).

**Aim:**

We aimed at exploring the effects of a structured program for the management of ICU patients, aimed at improving postdischarge HRQoL and reducing the risk of PICS.

**Methods:**

A total of 159 patients hospitalized in our ICU with a length of stay >72 hours were enrolled in an institutional management protocol including specific recommendations: adequate sedation and analgesia protocols, to ensure a valid delirium prevention strategy, and to provide a planned midterm after discharge. The main endpoint was the occurrence of PICS at the 6-month follow-up visitation, defined as an abnormal physical or mental score in the SF-12 questionnaire in the presence of clinical evidence of new or worsening impairment in physical, cognitive, or mental health status. An additional questionnaire was administered, to assess the effects of ICU-related memories.

**Results:**

Most patients positively rated their health at the 6-month follow-up and had no significant impairment in physical or mental health status. The mean normalized values of the physical and mental component of the SF-12 score were 46 ± 11 and 48 ± 14, suggesting a normal physical and mental health status in most patients. Twenty-nine patients (18.2%) showed evidence of PICS. Similar good results were found by the questionnaire of memories. In multivariable analysis, no variable was found to predict the risk of PICS in our population.

**Conclusion:**

In this real-world analysis that lacks a control group, patients who used a program aimed at minimizing the risk of HRQoL deterioration and PICS reported a good perception of their state of health with a relatively low prevalence of PICS.

## 1. Introduction

The primary role of the intensive care unit (ICU) is to treat life-threatening conditions in the acute phase, but also to minimize mortality and morbidity after discharge. To date, several studies previously focused on the risk of mortality after ICU hospitalization [1–4]. However, less attention has been given to morbidity and health-related quality of life (HRQoL) of patients who survive the initial critical event. It is known that, among patients discharged from an ICU, a significant proportion successively develop critical illness neuropathy, depression, clinical symptoms of posttraumatic stress disorder, and clinically overt cognitive deficits [5–9]. As a result, a multidimensional decline in HRQoL is known to occur in most patients after an ICU admission [10], in some cases determining the so-called post-intensive care syndrome (PICS)—a general condition characterized by a substantial deterioration in HRQoL that imposes relevant social costs related to the need of pharmacological and rehabilitative care [11, 12]. Several factors have been shown to increase the risk of HRQoL impairment among patients discharged from an ICU. Among these, an ICU stay lasting for more than 48 hours, mechanical ventilation, and the use of some sedative medications potentially associated with the onset of delirium seem to play a major role [8, 13–15]. The delirium, in particular, can generate the persistent mnemonic record of frightening and delusional memories, consolidating and strengthening the idea that the ICU environment was threatening, novice, and traumatic, and finally leading to a reduction in HRQoL [16, 17].

Based on these considerations, it can be hypothesized that a structured program based on appropriate sedation, analgesia, and delirium management during the ICU hospitalization phase, and including a plan for the midterm follow-up of patients after discharge, might be clinically useful to reduce the risk of HRQoL deterioration in these patients. The rationale of this is that the prevention of HRQoL impairment starts from a correct clinical management during hospitalization, but can also extend over the postdischarge period. In particular, a careful follow-up could help patients to correctly elaborate their memories and physicians to promptly identify patients with PICS symptoms and signs [18]. In this paper, we report the results of a real-world experience based on a program aimed at minimizing of the risk of HRQoL deterioration after ICU discharge, based on specific sedation, analgesia, and delirium protocols, and based on a predefined postdischarge follow-up plan.

## 2. Methods

In 2013, the medical staff of the Department of Anaesthesia and Intensive Care of the S. Maria Annunziata Hospital, Florence, Italy, established a structured program to reduce the risk of postdischarge HRQoL impairment for patients hospitalized in the local 6-bed ICU. For the purpose of this study, we considered for inclusion all patients who were hospitalized at our ICU during the first 2.5 years of employment of this structured program. Inclusion criteria were age > 18 years, length of stay in ICU > 72 hours, and adequate compliance to the follow-up program. Patients were excluded if they had severe cognitive impairment or relevant comorbidities with life expectancy < 6 months. A total of 70 consecutive patients meeting the same enrolling criteria, hospitalized during the last period before the starting of the program (2011-2012), were considered as a before-group. The program was based on four different issues: (1) adequate sedation protocol; (2) adequate analgesia protocol; (3) adequate delirium prevention strategy; (4) planned midterm follow-up after discharge.

### 2.1. Sedation Protocol

We decided to hinge our ICU sedation protocol on a systematic medication dose titration, based on the systematic assessment of sedation depth (every 8 hours) according to the Richmond Agitation Sedation Scale (RASS, target between −1 and +1, range −5 to +4) [19]. In this score, more negative values indicate deeper sedation and more positive scores indicate increasing agitation, with zero representing the status of calm and normal alertness. Our sedation protocol also included a daily interruption of sedation by temporary withdrawal of sedative agents, until the patient could give 3-4 simple answers or showed agitation. After this, the infusion was then re-established in a titrated form with the previous dose or half of the previous dose. We predefined some specific cases in which no interruption of sedation should be performed, including

- cardiovascular, respiratory, or neurological instability;
- patient receiving sedatives for control of seizures or alcohol abstinence;
- need of increase in the sedative dose for the control of agitation;
- patient receiving neuromuscular blockers;
- evidence of recent (<24 hours) myocardial infarction;
- evidence of intracranial pressure increase.

In addition to the daily awakening protocol, attention was given to keep the duration of long-term ventilation as short as possible. Following the current guidelines, propofol was identified as the first-choice medication for sedation in mechanically ventilated patients, and remifentanil as the reference treatment for sedation-based analgesia. Dexmedetomidine and midazolam were considered as second-choice medications in mechanically ventilated patients and as alternative options to propofol in subjects not submitted to mechanical ventilation, particularly in those with high risk of propofol infusion syndrome—for example, younger individuals and those with critical illness, use of vasopressors and glucocorticosteroids, or carbohydrate depletion due to liver disease or malnutrition. We also decided to avoid the routine use of benzodiazepines—unless already present in the usual home therapy—due to their association with increased risk of delirium and PICS [20, 21].

### 2.2. Analgesia Protocol

Our analgesia protocol was based on a systematic re-evaluation of the intensity of pain, performed three times a day at regular intervals using validated scales [22–24]. For patients able to communicate, the standard Visual Analogue Scale (VAS) was utilized. In this scale, patients mark their pain on a 100 mm line, with verbal descriptors at each end (0: no pain; 100: very severe pain). The score is obtained by measuring the distance in millimetres from the left end of the line. For patients unable to communicate, the Behavioural Pain Scale (BPS) was used. This scale is based on the clinical observation of facial expression, upper limb movements, and synchrony with mechanical ventilation. The BPS score ranges from 3 to 12, with values >6 indicating the need for pain management. Opioids and nonsteroidal anti-inflammatory drugs were identified as the reference medications for the treatment of pain.

### 2.3. Delirium Prevention

Our protocol for the diagnosis of delirium was based on the Confusion Assessment Method for Intensive Care (CM-ICU) [25]. We predefined a number of nonpharmacologic treatments for the prevention of delirium, including daily awakening trials, continuous reorienting of the patient to the environment, early mobilization, promotion of effective sleep/awake cycles, and minimization of continuous noise/stimulation at night. As already reported above, we decided to minimize the use of benzodiazepines for the prevention of delirium. A preference for haloperidol, hydroxyzine, and atypical antipsychotics such as olanzapine and quetiapine as first-line medications for the treatment of delirium was established [26].

### 2.4. Follow-Up

**Figure 1:**
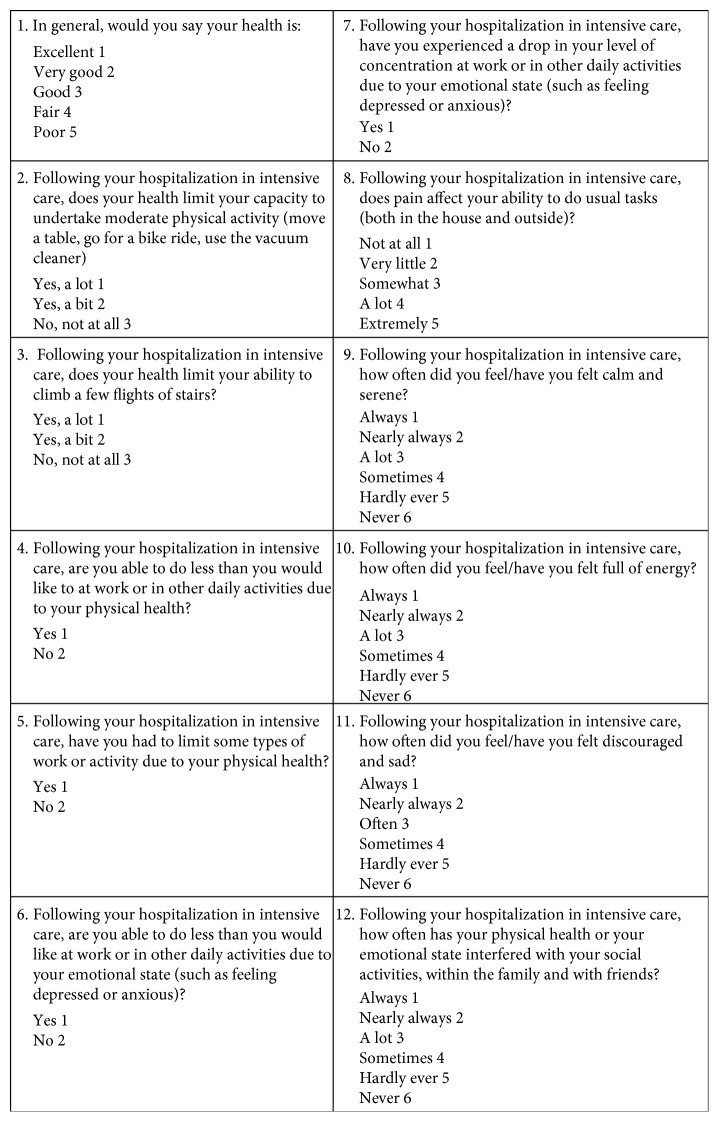
Short form-12 reduced.

**Figure 2:**
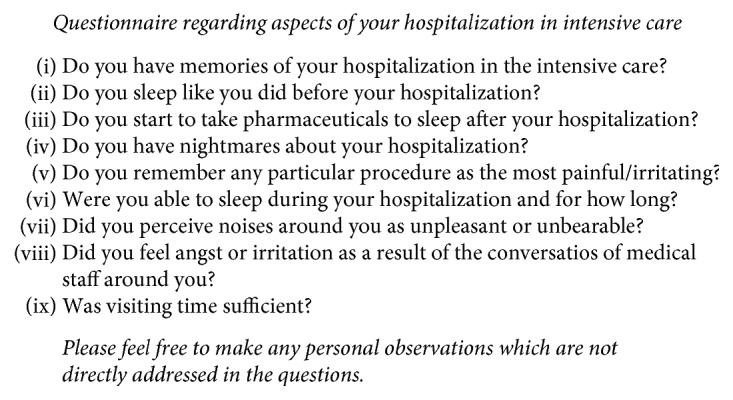
Questionnaire of memories.

The institutional program included a follow-up visitation after six months from discharge in all patients. In this visitation, along with a detailed clinical examination, patients were asked to complete two self-questionnaires. The first is the short form- (SF-) 12 questionnaire, a widely used multipurpose health status instrument aimed at assessing HRQoL and developed to provide a shorter but valid alternative to the SF-36 Health Survey (Figure 1) [27]. The SF-12 includes twelve questions, all selected from the SF-36, and provides easily interpretable weighted scales for both physical and mental health. In particular, the Physical Health Composite Score (PCS) and the Mental Health Composite Score (MCS) are calculated. Both PCS and MCS combine the answers given in the 12 items and compare them to a national norm, assumed to have a mean score of 50 and a SD of 10. In the resulting PCS and MCS, a score of 50 indicates the expected value according to age and higher values indicate highest levels of health. Therefore, in a given subject, a score higher or lower than 50 indicate better or lower health status than most individuals of similar age, with values between 30 and 70 representing the normality range and values <30 indicating impaired physical or mental status. Based on the results of the SF-12 and on the clinical evaluation at 6 months, patients were divided in those having and not having evidence of PICS. For the purpose of this study, patients with PICS were identified as those with PCS < 30 and/or MCS < 30, plus clinical evidence of new or worsening impairment in physical, cognitive, or mental health status, as assessed during the 6-month follow-up visitation [28]. The second questionnaire was a previously validated one specifically developed for the follow-up plan of ICU patients (Figure 2) [29]. Our follow-up protocol also included a patient's phone interview one year after discharge, where the SF-12 was again administered and where particular attention was given to potential problems identified during the first visitation.

### 2.5. Statistical Analysis

Data are shown as mean ± SD and number (percentage). The comparison between patients with and without PICS was performed by Student's *t*-test for independent groups for continuous variables and by the chi-square test or the Fisher exact test for categorical variables. Multivariable logistic regression was performed to assess independent predictors of PICS. Results were shown in terms of odds ratio and 95% confidence interval. The significance level was set at 0.05. All tests were two-tailed. Analyses were performed using the statistical package SPSS (Statistical Package for Social Sciences, Chicago, Illinois) for Windows, Release 15.0.

## 3. Results

**Figure 3:**
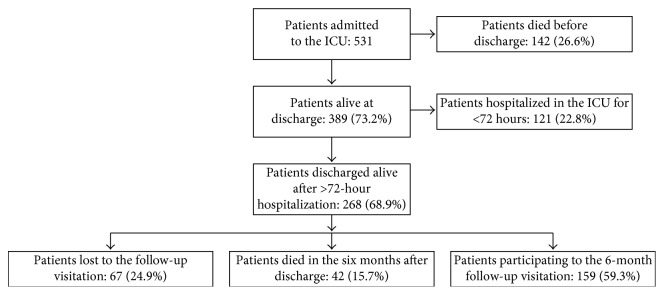
Flowchart for study population selection.

From January 2013 to May 2015, a total of 531 patients were admitted to the ICU. Of these, 142 (26.6%) died in the ICU or the ward before discharge, and 121 (22.8%) were hospitalized for a period of less than or equal to 72 hours. Among the remaining 268 patients who were discharged alive, 42 (15.7%) died in the six months after discharge. Also, 67 subjects did not present to the follow-up visitation, yielding an overall response rate of 75%. Therefore, the final study population included 159 patients (Figure 3). No significant differences in the main variables were observed between the group who completed the study protocol with the 6-month follow-up and those who were lost to follow-up.

Mean age of the patients included in the final study population was 63 ± 9 years. Seventy of them (44.0%) were women. The most common reasons for ICU admission included septic shock (*n*=30), coma (*n*=20), trauma (*n*=41), and perioperative complications in surgical patients with serious illness (*n*=42). Seventy-two patients (45.3%) required mechanical ventilation. By design, all patients participated to the 6-month follow-up visitation, and 42 of them (26.4%) accepted to participate to the one-year interview.

Most patients reported a good perception of their own state of health at the 6-month follow-up, with no significant limitations (Table 1). The mean normalized values of the PCS and MCS were 46 ± 11 and 48 ± 14, respectively, suggesting a normal physical and mental health status in most patients. A total of 29 patients (18.2%) were identified as having PICS, in most cases due to a simultaneous physical and mental impairment. With regard to the specific SF-12 items, nearly 70% of patients gave a positive judgment on their health (good, very good, or excellent) and complained no feelings of activity restrictions due to anxiety or depression, whereas more than half reported to feel serene or calm for most of the time and complained no significant feelings of activity restriction due to physical health. The reassessment of the SF-12 questionnaire by phone interview at one year provided similar results (Table 1, right columns).

Similar good results were provided by the questionnaire of memories (Table 2). About two-thirds of patients had memories of their hospitalization in the ICU, but less than 10% gave a negative judgement of these. Nearly 90% of patients claimed not to experience nightmares, and the majority stated that they were able to rest and to sleep like they did before their hospitalization. More than 80% of patients needed no medications to sleep after the ICU discharge and claimed that they did not perceive the noises around as unpleasant or intolerable.

The comparisons of main variables between patients with and without PICS at six months and that between the study population and the before-group are shown in Table 3. No significant differences in the main characteristics were observed between patients who developed and those who did not develop PICS. In a multivariable logistic analysis, none of the variables was associated with the risk of PICS. Similarly, no differences in the main variables were found between the study population and the before-group. Interestingly, considering the overall population of patients discharged alive, patients showed a slightly lower 6-month mortality (15.7% versus 27.1%, *p*=0.041) and a considerably lower prevalence of delirium (10.0% versus 55.7%, *p*=0.0001).

## 4. Discussion

In this study, we assessed the effects of a structured program aimed at minimizing the risk of HRQoL deterioration in patients discharged after an ICU hospitalization. In our population, most patients positively rated their health at the 6-month follow-up and had no significant impairment in physical or mental health status. About 20% of patients showed evidence of PICS. No variable was found to increase the risk of PICS in our population.

The prevalence of PICS found in our population was slightly lower than those reported in most studies on patients discharged from an ICU. In most of these, PICS has been reported to occur in 25% of ICU survivors, but some studies have reported an even higher incidence, occurring in more than three quarters of ICU survivors [12, 28, 30]. It has been shown that several factors—that is, false memories related to delirium or trauma, use of some medications such as benzodiazepines, circadian rhythm sleep disorders, and phases of impaired alertness during the hospitalization—can determine a worse perception of HRQoL after an ICU hospitalization, favouring the risk of PICS [31]. In this regard, affective memories such as fear, panic, anxiety, pain, depression, and more generally alterations in emotional state play a major role [12, 30, 32]. In addition, other ICU-related factors (e.g., deconditioning and severity of illness) and preexisting patient-related factors (e.g., dementia and comorbidities) can contribute to increase the risk of HRQoL worsening after an ICU hospitalization. In the BRAIN-ICU study, which assessed the predictors of neuropsychological dysfunction in >800 ICU survivors, 70% of patients developed delirium during the ICU stay, and longer delirium duration was associated with worse global cognitive and executive function at one year following discharge [33].

In our experience, the majority of our patients at the 6-month follow-up positively rated their health, complaining no feelings of activity restrictions due to anxiety, depression, or physical factors, and reported to feel serene or calm for most of the time. Most patients had no negative memories of their hospitalization in the ICU, claimed not to experience nightmares, were able to rest and to sleep as before their hospitalization without need of medications, and did not perceive the noises as unpleasant or intolerable. These findings could point out that, in the context of an ICU hospitalization, employing simple procedures and protocols aimed at minimizing the risk of postdischarge HRQoL deterioration might be useful in terms of perception of state of health at 6 months. In this regard, it is likely that the low prevalences of subjects with nightmares, anguish as a result of noise, and in general the absence of traumatic memories or delusions found in our analysis for most patients could partially reflect a positive impact on factual memories, as the absence of these is well known to be a strong risk factor for the consolidation of traumatic or false memories and for the onset of PICS [34–39]. It is also interesting that, in our population, none of the explored variables was associated with the risk of PICS. Although this finding could be affected by the relatively restrictive definition of PICS used in this study—based on the evidence of significantly abnormal SF-12 scores as an additional criterion to the clinical evidence of impaired physical, cognitive, or mental health status—such result may reflect the complex interaction of factors that are involved in determining the quality of life after an ICU hospitalization [32]. It is also interesting that only 45% of patients in our study population had a need for mechanical ventilation and that more than 40% of those who developed PICS did not have a need for mechanical ventilation. In this regard, it should be pointed out that sedation is often needed in subjects not submitted to mechanical ventilation, for example, patients with delirium, aggressive behaviours, and psychological disturbances; neurological patients with confusion/agitation and a relatively preserved Glasgow Coma Scale that does not compromise airway safet; and so on.

The present study has some limitations. As this was an analysis of a real-world structured protocol and not a trial, we did not have a control group. The final population included in this study represents only a proportion of the total population of patients who met the selection criteria at the time of the ICU hospitalization. This was the result of a 15% mortality during the first 6 months and a relatively high proportion of patients lost to follow-up or who did not present to the follow-up visit, which suggests that a significant amount of information was lost. Lastly, we were not able to compare the prevalence of PICS between the patients and our before-group, as this information was not available among subjects discharged before the implementation of the program.

In conclusion, after the implementation of a structured program aimed at minimizing the risk of HRQoL deterioration after ICU hospitalization, based on appropriate protocols for sedation, analgesia, and delirium prevention, and including a systematic midterm follow-up after discharge, most patients reported a good perception of their state of health, with a relatively low prevalence of PICS. Further studies are warranted to investigate the clinical utility of such an approach.

## Figures and Tables

**Table 1:** Results of the SF-12 questionnaire.

	6 months	%	1 year	%
*In general, would you say your health is:*				
Not noted	3	1.9	15	35.7
Good	68	42.8	13	31.0
Excellent	11	6.9	1	2.4
Very good	30	18.9	5	11.9
Fair	34	21.4	3	7.1
Poor	13	8.2	5	11.9
*Following your hospitalization in intensive care, does your health now limit you in moderate activities such as moving a table?*				
Not noted	4	2.5	15	35.7
No, not at all	82	51.6	13	31
Yes, a lot	32	20.1	7	16.7
Yes, a bit	41	25.8	7	16.7
*Following your hospitalization in intensive care, does your health limit your ability to climb a few flights of stairs?*				
Not noted	5	3.1	15	35.7
No, not at all	79	49.7	13	31
Yes, a lot	27	17	4	9.5
Yes, a bit	48	30.2	10	23.8
*Following your hospitalization in intensive care, have you had to limit some types of work or activity due to your physical health?*				
Not noted	5	3.1	16	38.1
No	81	50.9	12	28.6
Yes	73	45.9	14	33.3
*Following your hospitalization in intensive care, are you able to do less than you would like to at work or in other daily activities due to your physical health?*				
Not noted	5	3.1	16	38.1
No	83	52.2	11	26.2
Yes	71	44.7	15	35.7
*Following your hospitalization in intensive care, are you able to do less than you would like at work or in other daily activities due to your emotional state (such as feeling depressed or anxious)?*				
Not noted	5	3.1	16	38.1
No	104	65.4	15	35.7
Yes	50	31.4	11	26.2
*Following your hospitalization in intensive care, have you experienced a drop in your level of concentration in daily activities due to your emotional state?*				
Not noted	5	3.1	16	38.1
No	106	66.7	17	40.5
Yes	48	30.2	9	21.4
*Following your hospitalization in intensive care, does pain affect your ability to do usual tasks?*				
Not noted	5	3.1	16	38.1
Extremely	3	1.9	0	0
A lot	15	9.4	3	7.1
Very little	29	18.2	4	9.5
Not at all	72	45.3	10	23.8
Somewhat	35	22	9	21.4
*Following your hospitalization in intensive care, how often did you feel/have you felt calm and serene?*				
Not noted	4	2.5	16	38.1
Never	5	3.1	1	2.4
A lot	13	8.2	0	0.0
Hardly ever	6	3.8	1	2.4
Nearly always	59	37.1	13	31.0
Always	38	23.9	0	0.0
Sometimes	34	21.4	11	26.2
*Following your hospitalization in intensive care, how often did you feel/have you felt full of energy?*				
Not noted	7	4.4	16	38.1
Never	4	2.5	1	2.4
Nearly always	15	9.4	2	4.8
Hardly ever	26	16.4	9	21.4
A lot	39	24.5	3	7.1
Always	22	13.8	2	4.8
Sometimes	46	28.9	9	21.4
*Following your hospitalization in intensive care, how often did you feel/have you felt discouraged and sad?*				
Not noted	5	3.1	16	38.1
Never	34	21.4	3	7.1
A lot	11	6.9	3	7.1
Hardly ever	41	25.8	11	26.2
Nearly always	8	5	2	4.8
Always	8	5	0	0.0
Sometimes	52	32.7	7	16.7
*Following your hospitalization in intensive care, how often has your physical health or your emotional state interfered with your social activities?*				
Not noted	7	4.4	16	38.1
Never	38	23.9	2	4.8
A lot	2	1.3	1	2.4
Hardly ever	33	20.8	7	16.7
Nearly always	12	7.5	0	0.0
Always	10	6.3	1	2.4
Sometimes	57	35.8	15	35.7

**Table 2:** Results of the questionnaire of memories.

	Yes	No	I do not know	No answer
Do you have memories of your hospitalization in the intensive care?	107 (67.3%)	52 (32.7%)	—	0
If so, your judgment is positive?	54 (33.9%)	14 (8.8%)	22 (13.8%)	17 (15.9%)
Do you sleep like you did before your hospitalization?	119 (74.8%)	36 (22.6%)	—	4 (2.5%)
Did you start to take pharmaceuticals to sleep after your hospitalization?	19 (12.0%)	134 (84.3%)	—	6 (3.8%)
Do you have nightmares about your hospitalization?	13 (8.2%)	139 (87.4%)	—	7 (4.4%)
Were you able to sleep during your hospitalization and for how long?	91 (57.2%)	55 (34.6%)	—	13 (8%)
Did you perceive noises around you as unpleasant or unbearable?	18 (11.3%)	130 (81.7%)	—	11 (6.9%)
Did you feel angst or irritation as a result of the conversations of medical staff around you?	10 (6.3%)	129 (81.1%)	—	20 (12.6%)
Was visiting time sufficient?	98 (61. 7%)	27 (17.0%)	—	34 (21.4%)

**Table 3:** Main characteristics.

	PICS (*n*=29)	No PICS (*n*=130)	*p* value^∗^	Before- group (*n*=70)	*p* value^∗∗^
Age (years)	66 ± 7	63 ± 8	0.07	64 ± 8	0.21
Female gender (*n*)	15 (51.7%)	55 (42.3%)	0.36	30 (42.3%)	0.99
Need of ventilation support (*n*)	17 (58.6%)	55 (42.3%)	0.11	36 (51.4%)	0.48
Perioperative complications^a^ (*n*)	6 (20.7%)	20 (15.4%)	0.67	10 (14.3%)	0.84
Severe comorbidities^b^ (*n*)	3 (10.3%)	13 (10.0%)	0.78	7 (10.0%)	0.82
Septic shock^c^ (*n*)	5 (17.2%)	25 (19.2%)	0.80	15 (21.4%)	0.79
Coma^d^ (*n*)	4 (13.8%)	16 (12.3%)	0.76	7 (10.0%)	0.74
Trauma (*n*)	3 (10.3%)	38 (29.2%)	0.11	11 (15.7%)	0.13
Delirium (*n*)	27 (93.1%)	—	<0.0001	39 (55.7%)	<0.0001
Mortality (*n*)	20 (69.0%)	22 (16.9%)	<0.0001	19 (27.1%)	0.041

^∗^PICS versus no PICS. ^∗∗^Patients (pooled population with PICS and no PICS) versus controls. ^a^Severe bleeding, postoperative respiratory failure requiring ventilation, and haemodynamic instability. ^b^Defined as Charlson index ≥ 4. ^c^Diagnosed according to the 2016 Third International Consensus Definitions for Sepsis and Septic Shock. Secondary to polmonitis (*n*=8), peritonitis (*n*=8), central nervous system infection (*n*=6), urinary tract infection (*n*=4), cutaneous infection (*n*=3), and gastroenteritis (*n*=1). ^d^Defined as a score of −4 to −5 on the RASS scale.
